# Novel highly accelerated real-time CINE-MRI featuring compressed sensing with k-t regularization in comparison to TSENSE segmented and real-time Cine imaging

**DOI:** 10.1186/1532-429X-15-S1-P36

**Published:** 2013-01-30

**Authors:** Michaela Schmidt, Okan Ekinci, Jun Liu, Alban Lefebvre, Mariappan S Nadar, Edgar Mueller, Michael O Zenge

**Affiliations:** 1 MR PI, Siemens AG, Erlangen, Germany; 2 SCR, Siemens AG, Princeton, NY, USA; 3 CX CRM, Siemens AG, Erlangen, Germany

## Background

In patients with breath-holding difficulties or arrhythmia, real-time CINE-MRI is preferred over segmented acquisitions in one breath-hold. However, common real-time sequences require a deteriorating trade-off between spatial and temporal resolution. In the current work, highly accelerated real-time CINE-MRI which features compressed sensing with k t regularization [1] was evaluated against segmented and real-time imaging with TSENSE in healthy volunteers as a potential alternative providing both high spatial and temporal resolution in real time.

## Methods

Sparse and incoherent sampling was implemented in a bSSFP 2D CINE-MRI sequence and a compressed sensing image reconstruction program featuring k-t regularization was provided. Thirteen healthy volunteers (7m/6f, age 43±17y, BMI 24±6.6) underwent CMR imaging on a 1.5T system (MAGNETOM Aera, Siemens AG, Erlangen, Germany). 2-/3-/4-chamber as well as 3 short-axis views were acquired with a fixed temporal resolution of 33 ms but different net acceleration factors (NAF) and acquisition durations (acq) based on the used sequences:

(1) segmented TSENSE, NAF 2, (sTSENSE2), acq: 6 heartbeats

(2) segmented TSENSE, NAF 4, (sTSENSE4), acq: 3 heartbeats

(3) real-time TSENSE, NAF 4, (rtTSENSE4), acq: 1 heartbeat

(4) real-time compressed sensing, NAF 10.9, (rtCS11), acq: 1 heartbeat

The acquired (reconstructed) voxel sizes were 2.4 x 1.7 x 6 mm^3^ (1.7 x 1.7 x 6 mm^3^), except for rtTSENSE4 with 6.0 x 3.0 x 6 mm^3^ (3.0 x 3.0 x 6mm^3^). Image reconstruction was performed online. All images were qualitatively assessed by an experienced CMR reader on a five-point Likert scale (5-excellent, 1-non-diagnostic). Scoring was performed with respect to the overall image quality with focus on presence/severity of artifacts and the ability to visually assess global and regional myocardial function. A paired t-test was used to compare differences in image quality between the different sequences.

**Figure 1:**
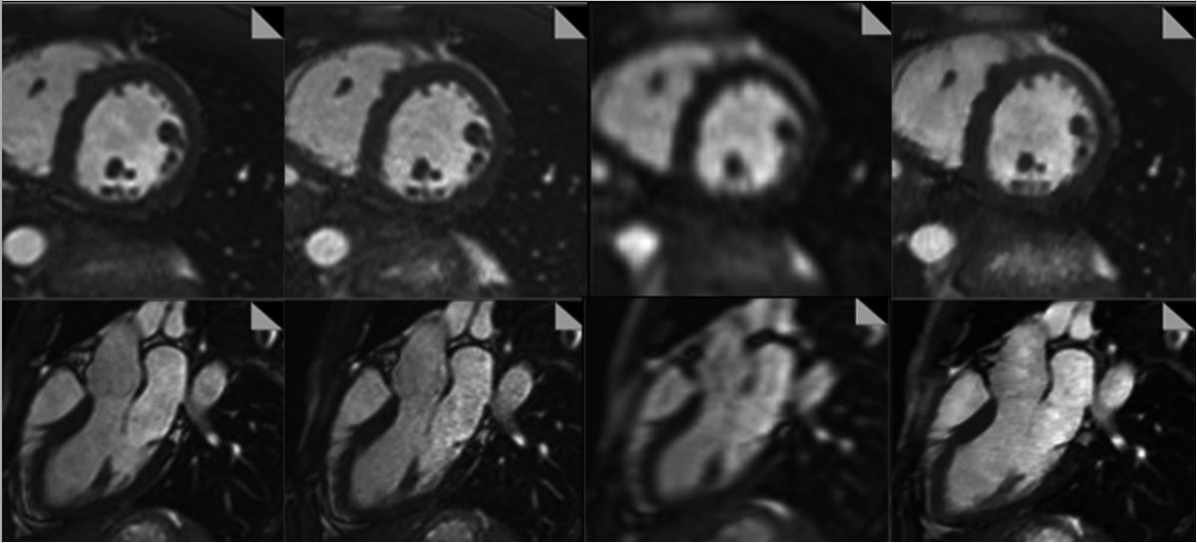
Example images of a short-axis and a 3-chamber view of volunteer 5 from left to right: sTSENSE2; sTSENSE4; rtTSENSE4; rtCS11

## Results

In all subjects, 2D datasets could be successfully acquired. The mean RR interval was 934±116 ms, three volunteers had sinus arrhythmia or extra systoles. Table 1 illustrates the results of the quality assessment. In terms of quality score, benchmark was set by sTSENSE2 (4.7±0.5). rtCS11 was significantly better than rtTSENSE4 (3.6±0.7 vs. 2.7±0.6, p<0.0001) and comparable to the quality of sTSENSE4 (3.9±0.5, p=0.004). Quality-relevant artifacts were rather noise-related in sTSENSE4 and contour- as well as flow-related in rtCS11.

**Table 1:** Quality assessment

	sTSENSE2	sTSENSE4	rtTSENSE4	rtCS11
Acq/temporal resolution	6hb/33 ms	3hb/33 ms	1hb/33 ms	1hb/33 ms
Spatial Resolution/SLT (mm)	2.4x1.7x6/6	2.4x1.7x6/6	6.0x3.0x3.0/6	2.4x1.7x6/6
Mean overall image quality	4.8±0.5	3.9±0.5	2.7±0.6	3.7±0.7
Comparison with rtCS11	p<0.001	p=0.004	p<0.001	

## Conclusions

As the image quality of rtCS11 was significantly better than in case of real-time TSENSE and close to that of sTSENSE4, the novel method may become a better alternative for the assessment of cardiac function in real time. Further studies in a clinical setting are required to assess the performance in challenging cases.

## Funding

Siemens AG
